# More similarity than difference: comparison of within- and between-sex variance in early adolescent brain structure

**DOI:** 10.21203/rs.3.rs-4947186/v1

**Published:** 2024-10-17

**Authors:** Carinna Torgerson, Katherine Bottenhorn, Hedyeh Ahmadi, Jeiran Choupan, Megan M. Herting

**Affiliations:** University of Southern California; University of Southern California; University of Southern California; University of Southern California; University of Southern California

## Abstract

**Background:**

Adolescent neuroimaging studies of sex differences in the human brain predominantly examine mean differences between males and females. This focus on between-groups differences without probing relative distributions and similarities may contribute to both conflation and overestimation of sex differences and sexual dimorphism in the developing human brain.

**Methods:**

We aimed to characterize the variance in brain macro- and micro-structure in early adolescence as it pertains to sex at birth using a large sample of 9–11 year-olds from the Adolescent Brain Cognitive Development (ABCD) Study (N=7,723). Specifically, for global and regional estimates of gray and white matter volume, cortical thickness, and white matter microstructure (i.e., fractional anisotropy and mean diffusivity), we examined: within- and between-sex variance, overlap between male and female distributions, inhomogeneity of variance via the Fligner-Killeen test, and an analysis of similarities (ANOSIM). For completeness, we examined these sex differences using both uncorrected (raw) brain estimates and residualized brain estimates after using mixed-effects modeling to account for age, pubertal development, socioeconomic status, race, ethnicity, MRI scanner manufacturer, and total brain volume, where applicable.

**Results:**

The overlap between male and female distributions was universally greater than the difference (overlap coefficient range: 0.585 – 0.985) and the ratio of within-sex and between-sex differences was similar (ANOSIM R range: −0.001 – 0.117). All cortical and subcortical volumes showed significant inhomogeneity of variance, whereas a minority of brain regions showed significant sex differences in variance for cortical thickness, white matter volume, fractional anisotropy, and mean diffusivity. Inhomogeneity of variance was reduced after accounting for other sources of variance. Overlap coefficients were larger and ANOSIM R values were smaller for residualized outcomes, indicating greater within- and smaller between-sex differences once accounting for other covariates.

**Conclusions:**

Reported sex differences in early adolescent human brain structure may be driven by disparities in variance, rather than binary, sex-based phenotypes. Contrary to the popular view of the brain as sexually dimorphic, we found more similarity than difference between sexes in all global and regional measurements of brain structure examined. This study builds upon previous findings illustrating the importance of considering variance when examining sex differences in brain structure.

## Plain English Summary

Brain imaging research has consistently revealed differences between males and females in the shape and size of adolescent brains. Studies usually compare the average male brain to the average female brain. However, brain structure varies greatly among individuals, even within the same sex. Without looking at both the variability within people of the same sex, and the degree of similarity between the sexes, it is unclear if separating adolescent brains into male and female categories will help us understand brain development. In this study, we looked at the overlap in brain structure among male and female youths (ages 9 to 11 years). We also compared variability between sexes and within each sex. Overall, we found that, there was more similarity than difference between male and female brains. The difference between any given male and any given female was similar to the difference between two individuals of the same sex. These findings suggest that, despite some small average differences, the brains of early adolescent males and females are more alike than different at ages 9–11 years.

## Background

Sexual dimorphism refers to traits with two distinct forms, each existing predominantly or exclusively among one sex, whereas sex differences describe traits that fall along a continuum, but exhibit a difference in mean or variability between males and females (1, 2). In the neuroscience literature, the conflation of the terms is exacerbated by researchers’ tendency to focus on mean sex differences. For example, when interpreting sex differences, the mean trait or phenotype is often generalized to the entire sex (i.e., “males have larger brains than females”) (3). In addition to differences attributable to differential expression of X- and Y-chromosome genes, the organizational-activational hypothesis posits that sex differences in exposure to steroid hormones during puberty cause both structural and functional sex differences in the brain and other non-gonadal tissues (4–6). This makes adolescence a crucial period of study for the development of sex differences in the brain.

Adolescent studies of sex differences in brain structure predominantly test for significant mean group differences between males and females (7–10). On average, regional cortical volumes are larger among male adolescents than among female adolescents (11, 12), as are a number of subcortical regions, including the putamen, pallidum, amygdala, thalamus, and cerebellum (12–14). However, some authors have reported greater whole-brain cortical thickness in adolescent females than in males (15), while others reported no sex differences (16–18). In addition to increased gray matter volume, male adolescents also display increased white matter volumes relative to female adolescents (19). Studies of fractional anisotropy (FA) and mean diffusivity (MD) - measures of white matter microstructure commonly used to study white matter development and integrity – have shown mixed results. For example, some studies report higher FA in male adolescents compared to females (20–23) while others report higher FA in female adolescents (24, 25). However, females enter puberty and reach maturity at younger ages than males (26). Similarly, measures of gray and white matter structure peak earlier in girls than in boys (27, 28). Therefore, it is important to account for differences in both maturation and chronological age when studying peripubertal development.

Despite relatively small effect sizes, numerous studies have concluded that these differences amount to sexual dimorphism in the developing brain (12, 29–33). This elevation of sex differences to sexual dimorphism inappropriately uses aggregate statistical results to infer the nature of inter-individual relationships, which is a form of ecological fallacy (34–36). For example, though males have - on average − 9–10% larger brains in adolescence (31, 37, 38), this statistic alone does not indicate that a randomly selected female is more likely than not to have a regional brain volume below a randomly selected male or below the population mean. Similarly, a mean sex difference is not sufficient evidence to claim that all females are more similar to each other than to any males. Such a comparison would require a deeper understanding of the dispersion of the data, particularly the relative within- and between-sex variance (39). Therefore, more nuanced statistical approaches are required to more fully contextualize the sex differences noted in the existing neuroimaging literature. In fact, in adults, overlap distribution statistics and formal analyses of similarity have shown extensive overlap between the distributions of MRI brain outcomes for each sex (N = 1,403; total age ranges 12–75 years) (40) and that brain metrics from two random individuals of the same sex differ as much as those from two random individuals of the opposite sex (3). These innovative statistical approaches challenge the narrative of “hard-wired” differences between “male brains” and “female brains” (41–52). However, similar research contextualizing sex differences in child and adolescent brains remains sparse.

Using the largest study of brain development - the Adolescent Brain Cognitive Development^℠^ Study (ABCD Study^®^) - we recently examined how sex and gender relate to gray matter macrostructure and white matter microstructure in a nationwide U.S. sample of 9–11 year-olds (23). We found that sex - but not felt-gender - was a significant predictor of early adolescent subcortical volume, cortical thickness, local gyrification index and white matter microstructure in the majority of regions examined. Furthermore, Wierenga, et al. (53, 54) previously found that male variability in the volumes of the hippocampus, pallidum, putamen, and cerebral gray and white matter was greater than female variability not only at the sample mean, but also at the extremes upper and lower ends of the distribution for children and adolescents. Building on this work, we examined inter-individual variability in brain development in the ABCD Study and found sex differences in the variability of the annualized percent change (55). Specifically, we reported greater male variability in white matter volumes and network connectivity, but greater female variability in the development of cortical macro- and micro-structure.

Consequently, this study aims to contextualize the cross-sectional relationship between mean group sex differences and inter-individual differences in brain structure in early adolescents ages 9 to 11 years old. Building upon our previous findings showing widespread, yet very small effect sizes (23), we aimed to further characterize within- and between-group differences, inhomogeneity between the sexes, overlap between the sexes, and conduct an analysis of similarity (ANOSIM) across various macro- and micro-structural brain metrics between males and females. Given large differences in overall head sizes and other potential confounders, we conducted our analyses on both raw (uncorrected) brain metrics and after adjusting for total brain volume (TBV) and other sociodemographic factors. Based on previous studies, we hypothesized that the variance between the male and female means would not exceed the within-sex variance, and that this would be true of more regions after adjusting for covariates. We expected inhomogeneity of variance between males and females, in line with previous research (53, 55). In terms of overlap, we hypothesized that we would find substantial overlap (i.e. greater than 50% overlap) of male and female distributions in all regions and measures examined, and that this overlap would be larger after adjusting for potential confounders, including TBV for volumetric outcomes.

## Methods

### Participants

This study utilized data collected as part of the larger ongoing Adolescent Brain Cognitive Development (ABCD) Study^®^, which involves 11,880 children at 21 different sites around the United States (56–58). The study included children from diverse geographic, demographic, and socioeconomic backgrounds (59, 60). Children with severe sensory, neurological, medical or intellectual limitations, lack of English proficiency or inability to complete an MRI scan were excluded from the ABCD Study (61). With respect to age, sex, and household size, the ABCD cohort closely matches the distribution of 9–11-year-olds in the American Community Survey, a large probability sample survey of U.S. households conducted annually by the U.S. Bureau of Census (60). Raw and minimally processed data are publicly available from the ABCD Study in service of increasing reproducibility. We utilized a combination of raw and tabulated questionnaire and neuroimaging data from the study baseline as obtained from the NDA 3.0 (raw T1 and T2 structural MRI files) and 4.0 (tabulated questionnaire and diffusion MRI) releases (NDA 3.0 and 4.0 data release 2021; https://dx.doi.org/10.15154/1523041). We chose to perform our own preprocessing for gray matter macrostructure using both T1w and T2w images to improve parcellation accuracy (23).

After obtaining the data, we implemented a series of quality control standards (Supplemental Fig. 1). Participants were excluded if their data were collected outside the 21 primary research sites, failed execution of the pre-processing or processing pipelines, failed to meet the raw or post-processing quality control standards of the ABCD consortium (58), or had incidental neurological findings noted by a radiologist (61). To reduce within-family correlation and meet statistical assumptions for independence, we decided to restrict our sample to one child per family (chosen randomly).

### Sex

The ABCD Study collects parent-reported sex assigned at birth. However, due to the multidimensional nature of sex, assignment at birth is not always an accurate reflection of chromosomal sex. Therefore, we also chose to use the frequency ratio of X and Y alleles to detect the presence of a Y chromosome and ascertain the genetic sex of participants. Children whose assigned sex and genetic sex did not match (n = 9) were excluded from the analysis.

### Neuroimaging Data

A harmonized data collection protocol was utilized across sites with either a Siemens, Phillips, or GE 3T MRI scanner. Motion compliance training, as well as real-time, prospective motion correction was used to reduce motion distortion (58). T1-weighted images were acquired using a magnetization-prepared rapid acquisition gradient echo (MPRAGE) sequence (TR = 2500, TE = 2.88, flip angle = 8) and T2-weighted images were obtained with fast spin echo sequence (TR = 3200, TE = 565, variable flip angle), with 176 slices with 1 mm^3^ isotropic resolution (57). Diffusion MRI data was acquired in the axial plane at 1.7 mm^3^ isotropic resolution with multiband acceleration factor 3. Ninety-six non-collinear gradient directions were collected with seven b0 images. Trained technicians inspected T1w, T2w, and dMRI images using a centralized quality control process in order to identify severe artifacts or irregularities (58).

To assess gray matter macrostructure, we obtained baseline T1w and T2w images from the ABCD 3.0 release (NDA 3.0 data release 2020; https://dx.doi.org/10.15154/1520591) and implemented the Human Connectome Project minimal preprocessing pipeline (62) at the Stevens Institute of Neuroimaging and Informatics. Regional parcellation and segmentation were then performed based on the Desikan-Killiany atlas in FreeSurfer 7.1.1 for each participant using T1w and T2w images (63). The primary outcomes of interest included gray matter volume, thickness, and white matter volume in 68 cortical regions, the volume of 20 subcortical regions, as well as FA and MD for 19 white matter tracts (64). For a complete list of regions by feature, please see Supplemental Table 1.

Tabulated white matter microstructure and demographic data from the baseline study visit were obtained from the 4.0 data release via the NIMH Data Archive (https://nda.nih.gov/abcd/; http://dx.doi.org/10.15154/1523041). ABCD diffusion processing employs five iterations of eddy current correction and robust tensor fitting to minimize gradient distortions and motion (58, 65). The b = 0 images are coarsely registered to a diffusion atlas before being registered to T1w images via mutual information. DMRI images are then resampled and registered using the transform from rigid registration of the T1w image to the diffusion atlas. Finally, the diffusion gradient matrix is adjusted for head rotation. Probabilistic atlas-based tractography is then performed with AtlasTrack using a priori tract location probabilities to inform fiber selection (65). For this study, we utilized the tabulated FA and MD data from the AtlasTrack fiber atlas. Specifically, we selected the fornix, cingulate cingulum, parahippocampal cingulum, uncinate fasciculus, superior longitudinal fasciculus, inferior longitudinal fasciculus, inferior fronto-occipital fasciculus, anterior thalamic radiations, corticospinal tracts, and corpus callosum as regions of interest (ROIs).

### Analyses

All statistical analyses were conducted in R (66) with the vegan (67; version 2.6.4), lme4 (68; version 1.1.35.1;), effectsize (69; version 0.8.8), and bayestestR (70; version 0.13.2) packages. We characterized variance and distributional overlap in brain outcomes between the sexes, investigated inhomogeneity of variance between the sexes with the Fligner-Killeen test, and implemented an analysis of similarities (ANOSIM). To ascertain whether variance, distributional overlap, and ANOSIM findings between the sexes were partially driven by additional variables, we repeated these analyses using residuals of brain outcomes after adjusting for additional variables (see details below).

For each ROI, we first compared the variance between group means to the within-sex variance for each ROI to determine whether the differences between sexes exceeded the differences within each sex for each ROI. To compare the within-sex variance of males and females for each ROI, we also calculated the coefficient of variation (CV), which accounts for potential scaling effects (71). We then examined inhomogeneity of variance between males and females via the Fligner-Killeen test, which compares the variances of two groups using a median-centered chi-square (χ^2^) test (72). We also calculated the overlap coefficient (OVL) for each ROI using the bayestestR package in R (70), which measures the percentage of the sample that falls within the overlap between two distributions. To complement these descriptive analyses, we conducted an analysis of similarities (ANOSIM) with Euclidean distances with the vegan package in R (67). ANOSIM is a non-parametric method for comparing groups of a single sample on the basis of pairwise, ranked distances to determine whether the between-group differences are greater than the within-group differences. Significance is determined with a series of permutations that incrementally reorder group membership and calculate the proportion of permutations with an R greater than or equal to the observed R. ANOSIM R statistics range from − 1 (all within-sex > between-sex ranked distances) to 1 (all between-sex > within-sex ranked distances) (also see Supplemental Table 2).

Residuals for each brain outcome were obtained from linear mixed modeling using the lme4 package in R (66, 68). To account for additional sources of neuroanatomical variance beyond sex alone as well as site effects, the models included several independent variables as fixed effects along with data collection site as a random effect (i.e. the nesting of subjects within sites) (Supplemental Table 3). Age was measured in months and rounded to the nearest whole month. Pubertal development was assessed using the parent-report version of the Pubertal Development Scale (PDS) and categorized as prepuberty, early puberty, mid puberty, late puberty, and post-puberty (73–76). Since few children in this age range were in late puberty or post-pubertal, we combined the mid, late, and post-puberty groups into a single category (mid/late puberty). We chose to include measures of race, ethnicity, and socioeconomic status in our models because human neurodevelopment is sensitive to various ecological factors which, due to systemic social injustice, are correlated with sociocultural variables, such as race, ethnicity, and socioeconomic status (77, 78). Youth race was collected via caregiver report and caregivers were encouraged to select all answers that applied. Where more than one race was selected, we categorized participants as multiracial Black (if one of their selections was “Black”) or multiracial non-Black. Due to low group numbers, we combined Asian Indian, Chinese, Filipino/a, Japanese, Korean, Vietnamese, Other Asian, American Indian/Native American, Alaska Native, Native Hawaiian, Guamanian, Samoan, other Pacific Islander, and “other race” into a single category (“other race”). Youth ethnicity was parent-reported as either Hispanic or non-Hispanic. To encapsulate socio-economic status, we included educational attainment, operationalized as the highest level of education achieved in the household, and binned into the following categories: less than high school diploma, high school diploma or GED, some college, bachelor’s degree, or postgraduate degree. Idiosyncrasies of different MRI software and hardware can also impact brain segmentation (79), so we also included scanner manufacturer (Philips, Siemens, or GE) as a covariate. Lastly, we chose to include TBV as a covariate in our models of regional volume to account for the relationship between regional and whole-brain volume (80). Although studies of white matter microstructure generally do not adjust for whole-brain volume (81, 82), our recent findings in the ABCD cohort suggest adjusting for TBV can influence reported sex differences in FA and MD as well (23). Therefore, we elected to include TBV as a fixed effect but to conduct an additional set of white matter sensitivity analyses using the residuals without TBV in the models. TBV was calculated by FreeSurfer, then scaled by the sample root-mean-square.

## Results

A full description of the final sample for the current study can be found in Table 1. After stringent data cleaning, our final sample closely matched the full ABCD Study sample in terms of sex, age, pubertal development, ethnicity, and parental education but differed significantly in terms of race.

### Global Brain Measures

**Figure 1.**
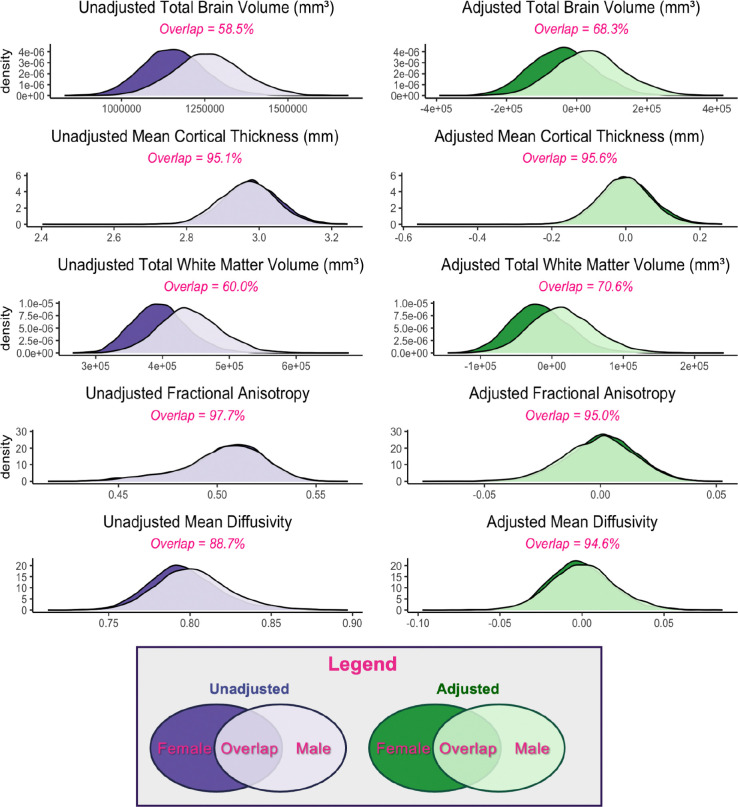
Overlap of global brain metrics in early adolescent males and females Density plots and overlap of whole-brain measurements for both the unadjusted (purple) and adjusted (i.e., residual estimates, green) estimates for male (light) and female (dark) adolescents. Please note that the x-axis and y-axis change between measures (i.e. between brain volume and FA) due to large differences in scale.

**Figure 2.**
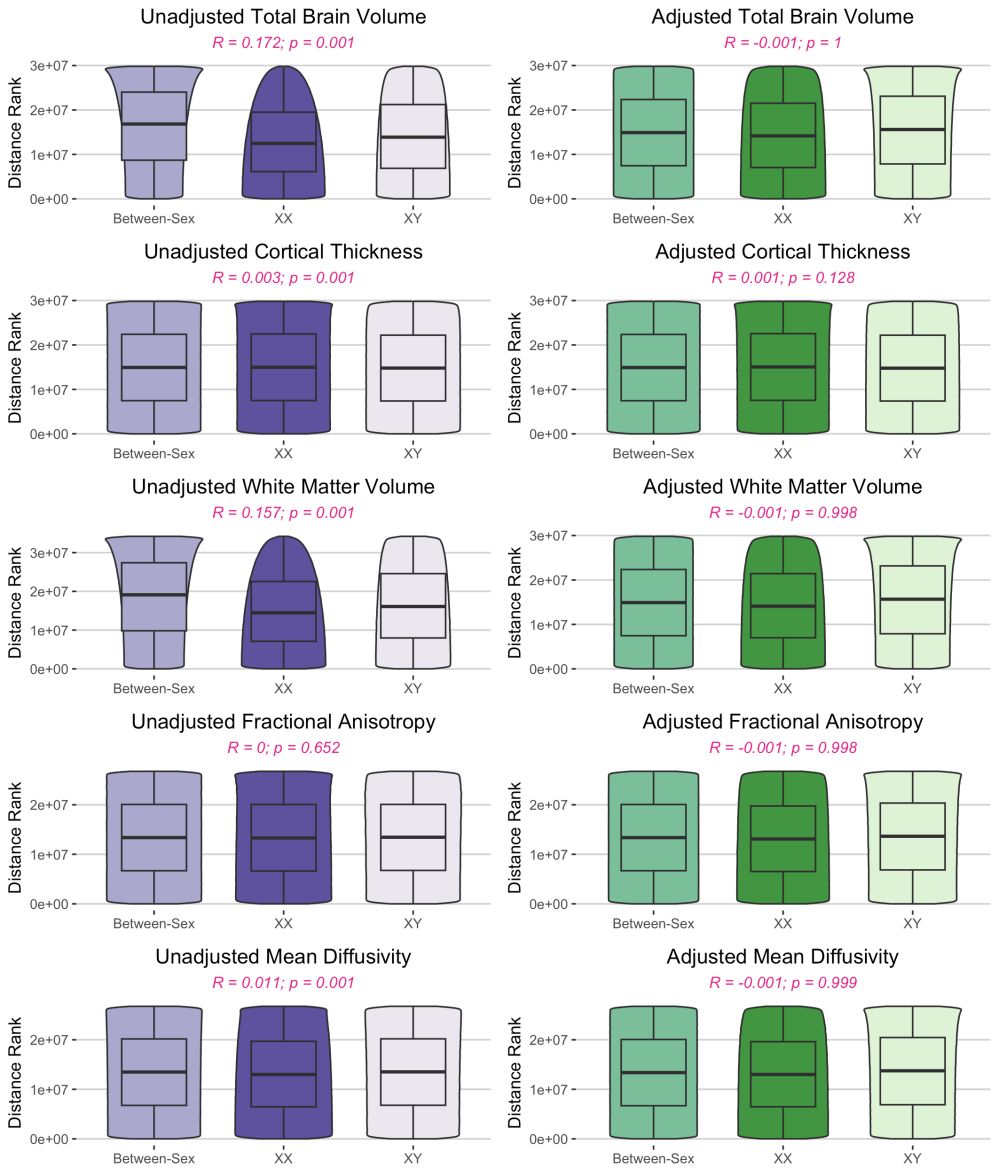
Similarity of within- and between-sex variance of global brain metrics in early adolescence. Violin plots of the within-sex and between-sex ranked distances from analysis of similarities (ANOSIM) test for both unadjusted (purple) and adjusted (residual estimates, green) as well as ANOSIM R statistic and FDR corrected p-values. An ANOSIM R statistic < 0.1 suggests that group ranks are similar.

In all global measures examined, within-sex variance exceeded between-sex variance, observed between the group means for male and female adolescents (Supplemental Table 4). For all whole-brain measures - both adjusted and unadjusted - the overlap between the male and female distributions was larger than the portions of the distribution unique to either sex (Fig. 1). In the unadjusted data, we observed inhomogeneity of variance in TBV and white matter volume, such that male variance was greater than female variance, although the CV of unadjusted global measures were very similar between males and females (Supplemental Table 4). After adjusting for additional variables, inhomogeneity of variance was significant for TBV, white matter volume, mean FA, and mean MD (Supplemental Table 4). ANOSIM tests showed that within-sex and between-sex distances were similar for all whole-brain measures examined (as denoted by ANOSIM R < 0.1), with the exception of TBV and total white matter volume, which were similar with some differences (i.e., R < 0.25) (Fig. 2; Supplemental Table 2). When adjusted values were used, all global measures showed similar variance between- and within-sex (Fig. 2).

### Regional Gray Matter and Subcortical Macrostructure

**Figure 3.**
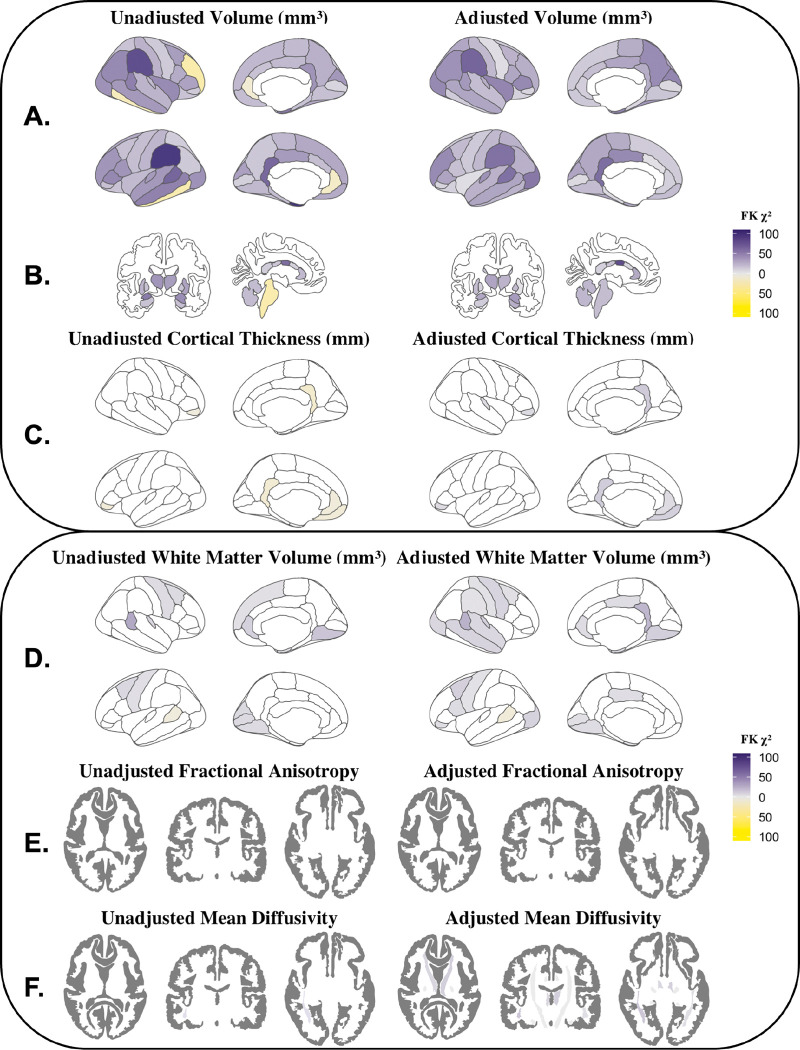
Unadjusted and adjusted inhomogeneity of variance between male and female adolescents for regional measures. A) cortical volumes, B) subcortical volumes, C) cortical thickness, D) white matter volumes, E) white matter fractional anisotropy (FA), and F) white matter mean diffusivity (MD). Colors reflect Fligner-Killeen χ2 statistic: purple denotes males > females; yellow denotes females > males.

**Figure 4.**
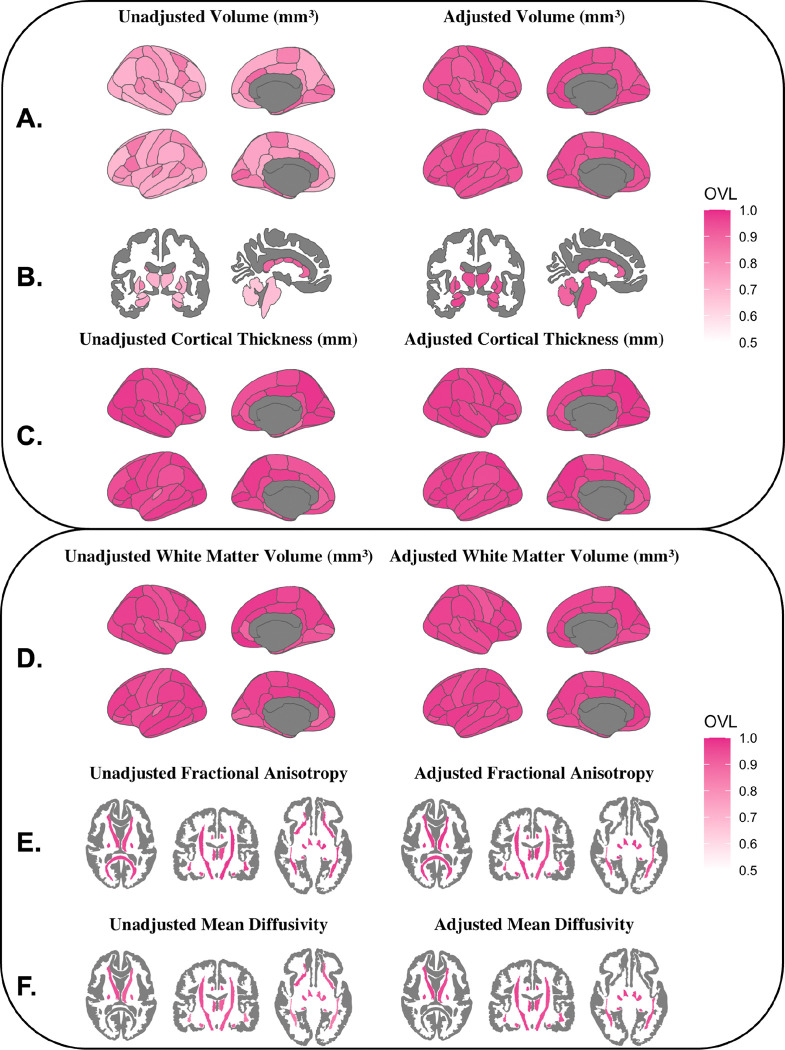
Unadjusted and adjusted overlap coefficients of male and female distributions for regional measures. A) cortical volumes, B) subcortical volumes, C) cortical thickness, D) white matter volumes, E) white matter fractional anisotropy (FA), and F) white matter mean diffusivity (MD). The overlap coefficient (OVL) compares the common area between two distributions to the unique variance and ranges from 0 (non-overlapping) to 1 (identical distributions).

**Figure 5.**
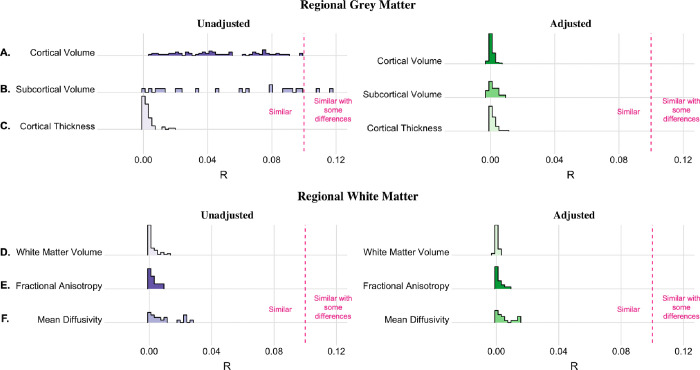
Distribution of Analysis of Similarities (ANOSIM) R statistics for unadjusted and adjusted regional measures. A) cortical volume, B) subcortical volume, C) cortical thickness, D) white matter volume, E) white matter fractional anisotropy (FA), and F) white matter mean diffusivity (MD). The ANOSIM R statistic ranges from − 1 to 1, with 0 indicating no disparity in the magnitude of between-group and within-group pairwise comparisons. Please note that these figures are trimmed to increase visibility and therefore, the x-axis does not display the full range of possible R statistics. The dashed pink line denotes the threshold for groups to be considered “similar with some differences” (see Supplemental Table 2).

The coefficients of variation for cortical volumes, subcortical volumes, and cortical thickness for male and female adolescents can be found in Supplemental Figs. 2–4. In all cortical gray matter and subcortical volumes as well as cortical thickness regions examined, the variance between group means was smaller than the within-sex variance using both the unadjusted and adjusted volumes (Supplemental Tables 5–7). For cortical and subcortical volumes, inhomogeneity of variance between sexes was significant in all regions, with greater variance among male adolescents than among female adolescents (Fig. 3A–B). The greatest sex differences in variance were seen in the supramarginal gyrus and central corpus callosum. In contrast to volume, female cortical thickness variance significantly exceeded male variance in the left superior frontal gyrus, left parahippocampal gyrus, and bilateral pericalcarine and lateral orbitofrontal cortices (Fig. 3C). Similar to the whole-brain analysis, the overlap coefficients were also large for both the unadjusted and adjusted cortical gray matter volumes, subcortical volumes, and cortical thickness (Fig. 4A–C). As expected, adjustment for TBV and other sources of variance led to an increase in the overlap of male and female regional cortical volumes (unadjusted: OVL range = 0.688–0.921, median = 0.788; adjusted: OVL range = 0.899–0.972, median = 0.939) and subcortical volumes (unadjusted: OVL range = 0.659–0.921, median = 0.749; adjusted: OVL range = 0.896–0.959, median = 0.939). Although the ANOSIM permutation tests were significant in 26 cortical and subcortical ROIs after FDR correction, the R statistic was consistently low (unadjusted: R statistic range: 0.0008–0.1171; median = 0.0446; adjusted: R statistic range: −0.0013–0.0086; median = 0.0001), indicating that the between-sex variance and within-sex variance were similar (Fig. 5A–B). The same pattern was found in cortical thickness, where 56% of regions were significant before adjustment, albeit with R statistic values reflective of no meaningful difference in rank between the groups (unadjusted: R statistic range − 0.0004–0.0194; median = 0.0013; adjusted: R statistic range − 0.0007– 0.0100; median = 0.0008) (Fig. 5C).

### Regional White Matter Volume

In all regions examined, the variance in white matter volumes between sexes was smaller than the within-sex variance (Supplemental Table 8, Supplemental Fig. 5). Adjustment increased the percentage of regions with significant sex differences in variance (unadjusted: *p* < 0.05 in 23.5% of regions; adjusted: *p* < 0.05 in 41.2% of regions) (Fig. 3D). Where significant, males showed greater regional variance than females except in the banks of the left superior temporal sulcus, where female variance exceeded male variance. Overlap coefficients were similar before and after adjustment (unadjusted: OVL range = 0.879–0.987, median = 0.963; adjusted: OVL range = 0.928–0.984, median = 0.963) (Fig. 4D). The ANOSIM results were significant (*p* < 0.05) in 25/68 (37%) regions after FDR correction, yet the magnitude of the R statistic indicated that within- and between-sex variances were similar (unadjusted: R statistic range − 0.0010–0.0132; median = 0.0002; adjusted: R statistic range − 0.0011–0.0031; median = −0.0002; Fig. 4D).

### White Matter Tract Microstructure

For both FA and MD the variance between the male and female mean values was universally smaller than the within-sex variance (Supplemental Tables 9–10; Supplemental Figs. 6–7). In unadjusted FA values, no tracts showed significant inhomogeneity of variance. After adjusting for covariates, FA variance in the corpus callosum and right superior longitudinal fasciculus was significantly greater among male youth compared to female youth (Fig. 3E). Before and after adjustment, male youth displayed significantly greater MD variance than female youth in 12/19 ROIs: the right corticospinal tracts, right uncinate fasciculus, corpus callosum, and bilaterally in the fornix, anterior thalamic radiations, superior longitudinal fasciculus, inferior longitudinal fasciculus, inferior fronto-occipital fasciculus, and superior longitudinal fasciculus (Fig. 3F). Substantial overlap was observed (Fig. 4E–F) in both the raw and adjusted FA (unadjusted: OVL range = 0.893–0.981, median = 0.959; adjusted: OVL range = 0.899–0.984, median = 0.963) and MD (unadjusted: OVL range = 0.829–0.967, median = 0.924; adjusted: OVL range = 0.928–0.977, median = 0.961). Similar to gray matter findings, the ANOSIM permutation tests found the ratio of between-sex to within-sex variance to be significantly different from 0 in many regions; however, the ANOSIM R statistic was small both before (unadjusted: R statistic range − 0.0008–0.0267; median = 0.0027) and after adjustment (adjusted: R statistic range − 0.0009–0.0156; median = 0.0018), suggesting similarity in rank distance between the groups (Fig. 5E–F).

## Discussion

This study contextualizes previous reports of widespread group mean sex differences previously reported in early adolescence (21, 23, 31, 83–86) by comparing the within- and between-sex variance as well as quantifying the neuroanatomical similarities between the sexes at ages 9 to 11 years old. In line with previous research in the developing brain (53, 55, 87), we detected significant inhomogeneity of variance between male and female youths. Moreover, we observed extensive overlap between male and female distributions and found between-sex and within-sex ranked differences to be similar in magnitude for all global and regional measures examined. We conclude that mean group sex differences in early adolescent brain structure are considerably smaller than the sex similarities, and therefore do not reflect distinct sex-based phenotypes (e.g., sexual dimorphism). Holistically, these results underscore the importance of accounting for within-group variance and inhomogeneity of variance when probing sex differences in brain morphology.

To assess similarity, we calculated the overlap (OVL) between male and female distributions in each global and regional measure. The OVL was invariably greater than 0.5, illustrating that across all structural metrics examined, more than half of all youths fell within the overlapping portion of the male and female distributions. In other words, there were substantial similarities between males and females throughout the brain. Similar results have been shown in adults, where “extensive overlap” has been reported between male and female distributions in all brain regions examined (40). While male and female total brain volume (TBV) distributions showed more similarity than difference (raw OVL = 0.585; corrected OVL = 0.682), TBV showed the least overlap between sex distributions of any measure examined, both before and after adjustment. This further supports its status as the largest and most replicable sex difference in pediatric brain structure (12,31,88–90). However, brain size is related to overall body size (91, 92), so this difference may simply be a reflection of overall body size differences between male and female adolescents. Unadjusted regional overlap was lower for cortical and subcortical volume than for cortical thickness, FA, and MD - which had median regional OVLs greater than 0.9 before adjustment. After adjustment, overlap increased in most regions - particularly for regional volumes - and a minimum of 89.6% of the data fell within the overlap between male and female distributions for all adjusted regional measures. These findings further demonstrate that the brains of male and female youth appear very similar after accounting for additional sources of variance in the data. Therefore, our results extend the conclusions of Joel et al. (2015) to early adolescents and reaffirm that human brain macrostructure does not exist in binary, sexually dimorphic categories associated with sex, nor does it appear to exist on a continuum between male and female extremes.

This work expands upon previous findings of sex differences in within-sex variability in childhood (53, 55). Wierenga et al. reported greater male variability in gray matter volume, whereas Bottenhorn et al. found greater male variability in white matter change over time, but greater female variability in cortical macro- and micro-structural change over time. After adjustment, we found significant sex differences in variance for TBV, average FA, average MD, and all regional volumes, with large inhomogeneity in the parietal lobe, basal ganglia, and limbic regions. Male variance exceeded female variance in all gray matter volume regions both before and after adjustment. Higher male variability in volume and diffusivity may be due, in part, to random X chromosome inactivation: heterozygous females express two different alleles of a single gene in a mosaic pattern throughout the brain, whereas homozygous females and males with a single X chromosome exhibit uniform expression (93, 94). Consequently, if two alleles of an X-chromosome gene have opposite effects, males and homozygous females will exhibit one of two extreme phenotypes, while heterozygous females will exhibit a mixed phenotype, decreasing the average trait variability among females. These results suggest that male structural variability is greater than female structural variability in gray matter volume and white matter microstructure, whereas female variability exceeds male variability in cortical thickness. Therefore, future research should examine the link between X-chromosome genes and regional gray matter volumes, while other sources of sex-related variance - such as estrogen and testosterone differences (95, 96), BMI (97), aerobic fitness (98, 99), or eating behaviors (100) - should be explored with regard to cortical thickness variance.

Many univariate methods of comparison (i.e., t-tests, ANOVA) rely on the assumption of homogeneity of variance. Consequently, such tests are inappropriate for comparing sexes on measures with significant inhomogeneity of variance between sexes, such as gray matter volume. Given the combination of large within-sex variance and high overlap between distributions of male and female youth, it is important to instead test whether between-sex differences surpass within-sex differences. Thus, we used ANOSIM to assess the relative magnitude of all pairwise differences between subjects and test for significant differences between the within-group and between-group pairings. Although permutation tests indicated that in some regions we could reject the null hypothesis (i.e., within-sex and between-sex variances do not differ), it is possible for a statistical result to be “significantly different from zero yet inconsequentially small” in a sufficiently large sample (101, 102). For example, in the adjusted data, ANOSIM indicated that between-sex pairings were significantly different from within-sex pairings in 33% of ROIs, yet the maximum observed ANOSIM R statistic in the corrected regional data was 0.0156 (adjusted R range: −0.0013–0.0156). ANOSIM R statistics less than 0.1 indicate that the size of the difference between two adolescents of the same sex is similar to the size of the difference between two adolescents of the opposite sex (3, 103–105). The fact that the results were significantly different from 0, but also very similar to 0 suggests that the sample size is sufficiently large to produce results with statistical significance but little practical or clinical significance. The ubiquity of the high overlap and low R statistic demonstrates that high similarity exists even in the measures with the highest mean sex differences. For instance, the effect size of sex for TBV (f^2^ = 0.243) would be considered medium-sized by Cohen’s standards (106) and “extremely above average” for the ABCD dataset (101, 107). Nonetheless, the TBV overlap was still greater than the difference (corrected OVL = 0.683) and the within-sex and between-sex differences were similar in size (corrected ANOSIM R statistic = 0.10). This highlights the fact that it is possible to have a relatively large, statistically significant sex effect even when subjects of the same sex differ about as much as subjects of different sexes. It is therefore critical for future analyses of sex to account for the mean-variance relationship and consider non-parametric methods that do not assume homogeneity of variance between sexes.

Taken together, these results contradict claims of sexual dimorphism in pediatric brain structure and contextualize the discussion of sex differences. This distinction between sexual dimorphism and sex differences is meaningful not just in theory, but also in practice. The putative sexual dimorphism of the developing brain has been cited in arguments for single-sex education (108–110) and as evidence in court cases regarding the rights of juveniles (111, 112). Yet, the large overlap between male and female distributions, small ratio of between-sex to within-sex differences, and significant inhomogeneity of variance reported here indicate that average pediatric sex differences are likely due to disparities in variability rather than two distinct phenotypes with a large mean difference. This lends credence to arguments that conventional methods for preclinical and clinical research of sex differences are not well-designed for application to personalized medicine and are insufficient to address health disparities between males and females (113–115). Future research designs should employ more robust statistical methods and focus on precise sex-linked variables, such as hormones, chromosomes, gene expression, body size and composition, or social determinants of health.

### Limitations

Due to the cross-sectional nature of this study and the narrow age range of the participants, our results are limited in scope. As such, they should not be assumed to generalize to brain structure in early childhood, later in adolescence, adulthood or to longitudinal trajectories of brain development. Instead, they offer an in-depth look at the neuroanatomy of children between 9 and 11 years old. Furthermore, although sex is multifaceted and encompasses multiple hormonal, genetic, and gross anatomical features, we chose to focus on the presence or absence of a Y chromosome for our operational definition of sex. Consequently, it is unclear to what extent factors like hormone levels, gene expression, or X-chromosome inactivation play a role in our results. Additionally, as a non-experimental study, we cannot provide evidence of a causal link between sex chromosomes and variance. Since few studies examine the influence of social and environmental factors on neuroanatomical sex differences, some authors instead use the term “sex/gender” (116, 117). While our previous work with data from the ABCD Study showed felt-gender did not explain a significant amount of variance in gray or white matter structure (23), we cannot rule out the possible influence of other sociocultural factors that may be correlated with sex.

Although this study discusses significance in terms of p-values (corrected for multiple comparisons), statisticians increasingly warn against dichotomous interpretations of results (i.e., “significant” or “nonsignificant”) (118, 119) and overreliance on statistical significance to infer practical significance (120, 121). The frequency of small but significant f^2^ and ANOSIM R statistics found in this study further suggest that in such a large, diverse sample, p-values may not be reliable indicators of practical significance. This underscores the danger of dichotomous interpretation of statistical tests in large samples. As such, the significance of the inhomogeneity of variance results should also be interpreted with caution.

Moreover, the results may not be directly comparable between brain regions or metrics with very different mean outcomes (i.e. cerebellum volume vs. pars orbitalis volume, average cortical thickness vs. average FA). While this issue is frequently circumvented with standardization, we did not use this technique because it would have altered the variance we sought to characterize. Scaling was similarly rejected because of the associated reduction in significant digits for some measures. For example, when large values (such as TBV in mm^3^) are reduced to a smaller value (such as TBV in m^3^), the loss of precision could lead to more ties when rank-ordering the pairwise distances, ultimately impacting the ANOSIM results. Therefore, because of the regional differences in scale and the intrinsic link between the mean and variance, caution is urged when comparing results between different brain region outcomes.

## Conclusions

Early adolescent male and female brains are more similar than they are different. Due to high within-sex variability, the distributions of males and females have more overlap than difference on all measures of global and regional gray and white matter structure examined. Although male and female adolescents exhibited significant inhomogeneity of neuroanatomical variance, ANOSIM showed that within-sex and between-sex differences were similar in size. Overall, these results illustrate that sex differences in early adolescent brain structure do not amount to qualitative differences (e.g., sexual dimorphism), and that quantitative differences between sexes are likely too small to be practically meaningful compared with individual variability.

## Figures and Tables

**Table 1:** Demographic comparison between all ABCD Study subjects and the study sample.

	ABCD Cohort (N = 11,876)	Study Sample (N = 7,723)
**Sex**		
Female	5680 (47.8%)	3714 (48.1%)
Male	6196 (52.2%)	4009 (51.9%)
**Age (months)**		
Mean (SD)	119 (7.50)	119 (7.44)
Median [Min, Max]	119 [107, 133]	119 [107, 133]
**Pubertal Development**		
Pre	5837 (49.1%)	3938 (51.0%)
Early	2713 (22.8%)	1860 (24.1%)
Mid/Late	2854 (24.0%)	1925 (24.9%)
Missing	472 (4.0%)	0 (0%)
**Race** *		
White	7517 (63.3%)	5146 (66.6%)
Black	1868 (15.7%)	1062 (13.8%)
Multiracial (Black)	649 (5.5%)	408 (5.3%)
Multiracial (Non-Black)	785 (6.6%)	516 (6.7%)
Other^a^	874 (7.4%)	591 (7.7%)
Missing	183 (1.5%)	0 (0%)
**Ethnicity**		
Non-Hispanic	9312 (78.4%)	6147 (79.6%)
Hispanic	2411 (20.3%)	1576 (20.4%)
Missing	153 (1.3%)	0 (0%)
**Parent Education** *		
< High School Diploma	578 (4.9%)	296 (3.8%)
HS Diploma or GED	1110 (9.3%)	603 (7.8%)
Some College	3058 (25.7%)	1970 (25.5%)
Bachelor	3010 (25.3%)	2021 (26.2%)
Post Graduate Degree	4041 (34.0%)	2833 (36.7%)
Missing	79 (0.7%)	0 (0%)

* Difference between the research sample and the ABCD Study sample is statistically significant at the p <0.05 level.

a The “Other” race/ethnicity category includes participants who were parent-identified as Asian Indian, Chinese, Fiiipino/a, Japanese, Korean, Vietnamese, Other Asian, American Indian/Native American, Alaska Native, Native Hawaiian, Guamanian, Samoan, Other Pacific Islander, or Other Race

## Data Availability

Data from the ABCD Study Data Release are available for access through the NIMH Data Archive (NDA 3.0 data release 2020: https://doi.org/10.15154/1520591; NDA 4.0 data release 2021; https://doi.org/10.15154/1523041). Only researchers with an approved NDA Data Use Certification (DUC) can obtain ABCD Study data.

## Highlights

- High male/female overlap is ubiquitous across all brain features in early adolescence
- Male variance exceeded female variance for global and regional brain volumes
- Between- and within-sex differences were similar in magnitude for all features

## References

[1] DeCasien AR, Guma E, Liu S, Raznahan A. Sex differences in the human brain: a roadmap for more careful analysis and interpretation of a biological reality. Biol Sex Differ. 2022;13(1):43. 35883159 10.1186/s13293-022-00448-w PMC9327177

[2] McCarthy MM, Arnold AP, Ball GF, Blaustein JD, De Vries GJ. Sex differences in the brain: the not so inconvenient truth. J Neurosci. 2012;32(7):2241–7. 22396398 10.1523/JNEUROSCI.5372-11.2012 PMC3295598

[3] Sanchis-Segura C, Aguirre N, Cruz-Gómez ÁJ, Félix S, Forn C. Beyond sex prediction: Estimating and interpreting multivariate sex differences and similarities in the brain. NeuroImage. 2022;257:119343. 35654377 10.1016/j.neuroimage.2022.119343

[4] Arnold AP. The organizational–activational hypothesis as the foundation for a unified theory of sexual differentiation of all mammalian tissues. Horm Behav. 2009;55(5):570–8. 19446073 10.1016/j.yhbeh.2009.03.011 PMC3671905

[5] McCarthy MM, Wright CL, Schwarz JM. New tricks by an old dogma: mechanisms of the organizational/activational hypothesis of steroid-mediated sexual differentiation of brain and behavior. Horm Behav. 2009;55(5):655–65. 19682425 10.1016/j.yhbeh.2009.02.012 PMC2742630

[6] Schulz KM, Molenda-Figueira HA, Sisk CL. Back to the Future: The Organizational-Activational Hypothesis Adapted to Puberty and Adolescence. Horm Behav. 2009;55(5):597–604. 19446076 10.1016/j.yhbeh.2009.03.010 PMC2720102

[7] Giedd JN, Raznahan A, Mills KL, Lenroot RK. Magnetic resonance imaging of male/female differences in human adolescent brain anatomy. Biol Sex Differ. 2012;3(1):1–9. 22908911 10.1186/2042-6410-3-19 PMC3472204

[8] Giedd JN, Denker AH. The Adolescent Brain: Insights from Neuroimaging. In: Bourguignon JP, Carel JC, Christen Y, editors. Brain Crosstalk in Puberty and Adolescence [Internet]. Cham: Springer International Publishing; 2015 [cited 2022 Dec 28]. pp. 85–96. (Research and Perspectives in Endocrine Interactions). 10.1007/978-3-319-09168-6_7

[9] Kaczkurkin AN, Raznahan A, Satterthwaite TD. Sex differences in the developing brain: insights from multimodal neuroimaging. Neuropsychopharmacology. 2019;44(1):71–85. 29930385 10.1038/s41386-018-0111-z PMC6235840

[10] Lenroot RK, Giedd JN. Sex differences in the adolescent brain. Brain Cogn. 2010;72(1):46–55. 19913969 10.1016/j.bandc.2009.10.008 PMC2818549

[11] Gennatas ED, Avants BB, Wolf DH, Satterthwaite TD, Ruparel K, Ciric R, Age-Related Effects and Sex Differences in Gray Matter Density, Volume, Mass, and Cortical Thickness from Childhood to Young Adulthood. J Neurosci. 2017;37(20):5065–73. 28432144 10.1523/JNEUROSCI.3550-16.2017 PMC5444192

[12] Paus T, Nawaz-Khan I, Leonard G, Perron M, Pike GB, Pitiot A, Sexual dimorphism in the adolescent brain: role of testosterone and androgen receptor in global and local volumes of grey and white matter. Horm Behav. 2010;57(1):63–75. 19703457 10.1016/j.yhbeh.2009.08.004

[13] Adeli E, Zhao Q, Zahr NM, Goldstone A, Pfefferbaum A, Sullivan EV, Deep learning identifies morphological determinants of sex differences in the pre-adolescent brain. NeuroImage. 2020;223:117293. 32841716 10.1016/j.neuroimage.2020.117293 PMC7780846

[14] Paus T. Chapter 2 - Sex differences in the human brain: A developmental perspective. In: Savic I, editor. Progress in Brain Research [Internet]. Elsevier; 2010 [cited 2023 Jul 29]. pp. 13–28. (Sex Differences in the Human Brain, their Underpinnings and Implications; vol. 186). https://www.sciencedirect.com/science/article/pii/B9780444536303000026 21094883 10.1016/B978-0-444-53630-3.00002-6

[15] Zhou D, Lebel C, Treit S, Evans A, Beaulieu C. Accelerated longitudinal cortical thinning in adolescence. NeuroImage. 2015;104:138–45. 25312772 10.1016/j.neuroimage.2014.10.005

[16] Bramen JE, Hranilovich JA, Dahl RE, Chen J, Rosso C, Forbes EE, Sex Matters during Adolescence: Testosterone-Related Cortical Thickness Maturation Differs between Boys and Girls. PLoS ONE. 2012;7(3):e33850. 22479458 10.1371/journal.pone.0033850 PMC3315517

[17] Menary K, Collins PF, Porter JN, Muetzel R, Olson EA, Kumar V, Associations between cortical thickness and general intelligence in children, adolescents and young adults. Intelligence. 2013;41(5):597–606. 24744452 10.1016/j.intell.2013.07.010 PMC3985090

[18] Vijayakumar N, Allen NB, Youssef G, Dennison M, Yücel M, Simmons JG, Brain development during adolescence: a mixed-longitudinal investigation of cortical thickness, surface area, and volume. Hum Brain Mapp. 2016;37(6):2027–38. 26946457 10.1002/hbm.23154 PMC6867680

[19] Pfefferbaum A, Rohlfing T, Pohl KM, Lane B, Chu W, Kwon D, Adolescent Development of Cortical and White Matter Structure in the NCANDA Sample: Role of Sex, Ethnicity, Puberty, and Alcohol Drinking. Cereb Cortex. 2016;26(10):4101–21. 26408800 10.1093/cercor/bhv205 PMC5027999

[20] Herting MM, Maxwell EC, Irvine C, Nagel BJ. The Impact of Sex, Puberty, and Hormones on White Matter Microstructure in Adolescents. Cereb Cortex N Y NY. 2012;22(9):1979–92. 10.1093/cercor/bhr246 PMC3412439 22002939

[21] Lawrence KE, Abaryan Z, Laltoo E, Hernandez LM, Gandal MJ, McCracken JT, White matter microstructure shows sex differences in late childhood: Evidence from 6797 children. Hum Brain Mapp. 2023;44(2):535–48. 36177528 10.1002/hbm.26079 PMC9842921

[22] Pohl KM, Sullivan EV, Rohlfing T, Chu W, Kwon D, Nichols BN, Harmonizing DTI Measurements across Scanners to Examine the Development of White Matter Microstructure in 803 Adolescents of the NCANDA Study. NeuroImage. 2016;130:194–213. 26872408 10.1016/j.neuroimage.2016.01.061 PMC4808415

[23] Torgerson C, Ahmadi H, Choupan J, Fan CC, Blosnich JR, Herting MM. Sex, gender diversity, and brain structure in early adolescence. Hum Brain Mapp. 2024;45(5):e26671. 38590252 10.1002/hbm.26671 PMC11002534

[24] Bava S, Boucquey V, Goldenberg D, Thayer RE, Ward M, Jacobus J, Sex Differences in Adolescent White Matter Architecture. Brain Res. 2011;1375:41–8. 21172320 10.1016/j.brainres.2010.12.051 PMC3035918

[25] Schmithorst VJ, Holland SK, Dardzinski BJ. Developmental differences in white matter architecture between boys and girls. Hum Brain Mapp. 2007;29(6):696–710. 10.1002/hbm.20431 PMC2396458 17598163

[26] Brix N, Ernst A, Lauridsen LLB, Parner E, Støvring H, Olsen J, Timing of puberty in boys and girls: A population-based study. Paediatr Perinat Epidemiol. 2019;33(1):70–8. 30307620 10.1111/ppe.12507 PMC6378593

[27] Raznahan A, Shaw P, Lalonde F, Stockman M, Wallace GL, Greenstein D, How Does Your Cortex Grow? J Neurosci. 2011;31(19):7174–7. 21562281 10.1523/JNEUROSCI.0054-11.2011 PMC3157294

[28] Simmonds D, Hallquist MN, Asato M, Luna B. Developmental Stages and Sex Differences of White Matter and Behavioral Development through Adolescence: A Longitudinal Diffusion Tensor Imaging (DTI) Study. NeuroImage. 2014;92:356–68. 24384150 10.1016/j.neuroimage.2013.12.044 PMC4301413

[29] Brennan D, Wu T, Fan J. Morphometrical Brain Markers of Sex Difference. Cereb Cortex. 2021;31. 10.1093/cercor/bhab037 33774662

[30] Herting MM, Maxwell EC, Irvine C, Nagel BJ. The Impact of Sex, Puberty, and Hormones on White Matter Microstructure in Adolescents. Cereb Cortex. 2012;22(9):1979–92. 22002939 10.1093/cercor/bhr246 PMC3412439

[31] Lenroot RK, Gogtay N, Greenstein DK, Wells EM, Wallace GL, Clasen LS, Sexual dimorphism of brain developmental trajectories during childhood and adolescence. NeuroImage. 2007;36(4):1065–73. 17513132 10.1016/j.neuroimage.2007.03.053 PMC2040300

[32] Seunarine KK, Clayden JD, Jentschke S, Munoz M, Cooper JM, Chadwick MJ, Sexual dimorphism in white matter developmental trajectories using tract-based spatial statistics. Brain Connect. 2016;6(1):37–47. 26446207 10.1089/brain.2015.0340 PMC4744889

[33] Yang X, Li A, Li L, Li T, Li P, Liu M. Multimodal Image Analysis of Sexual Dimorphism in Developing Childhood Brain. Brain Topogr. 2021;34(3):257–68. 33630209 10.1007/s10548-021-00823-7

[34] Gnaldi M, Tomaselli V, Forcina A. Ecological fallacy and covariates: new insights based on multilevel modelling of individual data. Int Stat Rev. 2018;86(1):119–35.

[35] Nieri M, Clauser C, Pagliaro U, PiniPrato G. Individual patient data: A criterion in grading articles dealing with therapy outcomes. J Evid Based Dent Pract. 2003;3(3):122–6.

[36] Paik M. A graphic representation of a three-way contingency table: Simpson’s paradox and correlation. Am Stat. 1985;39(1):53–4.

[37] Giedd JN, Castellanos FX, Rajapakse JC, Vaituzis AC, Rapoport JL. Sexual dimorphism of the developing human brain. Prog Neuropsychopharmacol Biol Psychiatry. 1997;21(8):1185–201. 9460086 10.1016/s0278-5846(97)00158-9

[38] Giedd JN, Raznahan A, Alexander-Bloch A, Schmitt E, Gogtay N, Rapoport JL. Child Psychiatry Branch of the National Institute of Mental Health Longitudinal Structural Magnetic Resonance Imaging Study of Human Brain Development. Neuropsychopharmacology. 2015;40(1):43–9. 25195638 10.1038/npp.2014.236 PMC4262916

[39] Warton DI, Hui FKC. The central role of mean-variance relationships in the analysis of multivariate abundance data: a response to Roberts (2017). Methods Ecol Evol. 2017;8(11):1408–14.

[40] Joel D, Berman Z, Tavor I, Wexler N, Gaber O, Stein Y, Sex beyond the genitalia: The human brain mosaic. Proc Natl Acad Sci. 2015;112(50):15468–73. 26621705 10.1073/pnas.1509654112 PMC4687544

[41] Amen DG. Unleash the power of the female brain: Supercharging yours for better health, energy, mood, focus, and sex. Harmony; 2013.

[42] Baron-Cohen S. The essential difference: Male and female brains and the truth about autism. Basic Books; 2009.

[43] Blum D. Sex on the brain: The biological differences between men and women. Penguin; 1998.

[44] Brizendine L. The female brain. Broadway Books; 2006.

[45] Brizendine L. The Upgrade: How the Female Brain Gets Stronger and Better in Midlife and Beyond. Hay House, Inc; 2022.

[46] Darlington CL. The female brain. CRC; 2009.

[47] Gurian M. Boys and girls learn differently! A guide for teachers and parents. Wiley; 2010.

[48] Gurian M, Stevens K. How boys learn. Educ Horiz. 2006;84(2):87–93.

[49] James AN. Teaching the female brain: How girls learn math and science. Corwin; 2009.

[50] Lundin M. Female Brain Gone Insane: An Emergency Guide for Women who Feel Like They are Falling Apart. Health Communications, Inc.; 2009.

[51] McKay S. The Women’s Brain Book: The neuroscience of health, hormones and happiness. Hachette UK; 2018.

[52] Schulz ML. The new feminine brain: How women can develop their inner strengths, genius, and intuition. Simon and Schuster; 2005.

[53] Wierenga LM, Sexton JA, Laake P, Giedd JN, Tamnes CK, Pediatric Imaging N. A key characteristic of sex differences in the developing brain: greater variability in brain structure of boys than girls. Cereb Cortex. 2018;28(8):2741–51. 28981610 10.1093/cercor/bhx154 PMC6041809

[54] Wierenga LM, Doucet GE, Dima D, Agartz I, Aghajani M, Akudjedu TN, Greater male than female variability in regional brain structure across the lifespan. Hum Brain Mapp. 2022;43(1):470–99. 33044802 10.1002/hbm.25204 PMC8675415

[55] Bottenhorn KL, Cardenas-Iniguez C, Mills KL, Laird AR, Herting MM. Profiling intra- and inter-individual differences in brain development across early adolescence. NeuroImage. 2023;279:120287. 37536527 10.1016/j.neuroimage.2023.120287 PMC10833064

[56] ABCD Study [Internet]. 2022 [cited 2022 Apr 6]. ABCD Study. https://abcdstudy.org/about/

[57] Casey BJ, Cannonier T, Conley MI, Cohen AO, Barch DM, Heitzeg MM, The adolescent brain cognitive development (ABCD) study: imaging acquisition across 21 sites. Dev Cogn Neurosci. 2018;32:43–54. 29567376 10.1016/j.dcn.2018.03.001 PMC5999559

[58] Hagler DJ, Hatton S, Cornejo MD, Makowski C, Fair DA, Dick AS, Image processing and analysis methods for the Adolescent Brain Cognitive Development Study. NeuroImage. 2019;202:116091. 31415884 10.1016/j.neuroimage.2019.116091 PMC6981278

[59] Garavan H, Bartsch H, Conway K, Decastro A, Goldstein RZ, Heeringa S, Recruiting the ABCD sample: Design considerations and procedures. Dev Cogn Neurosci. 2018;32:16–22. 29703560 10.1016/j.dcn.2018.04.004 PMC6314286

[60] Heeringa SG, Berglund PA. A guide for population-based analysis of the Adolescent Brain Cognitive Development (ABCD) Study baseline data. BioRxiv. 2020.

[61] Li Y, Thompson WK, Reuter C, Nillo R, Jernigan T, Dale A, Rates of incidental findings in brain magnetic resonance imaging in children. JAMA Neurol. 2021;78(5):578–87. 33749724 10.1001/jamaneurol.2021.0306 PMC7985817

[62] Glasser MF, Sotiropoulos SN, Wilson JA, Coalson TS, Fischl B, Andersson JL, The minimal preprocessing pipelines for the Human Connectome Project. NeuroImage. 2013;80:105–24. 23668970 10.1016/j.neuroimage.2013.04.127 PMC3720813

[63] Desikan RS, Ségonne F, Fischl B, Quinn BT, Dickerson BC, Blacker D, An automated labeling system for subdividing the human cerebral cortex on MRI scans into gyral based regions of interest. NeuroImage. 2006;31(3):968–80. 16530430 10.1016/j.neuroimage.2006.01.021

[64] Hagler DJ Jr, Ahmadi ME, Kuperman J, Holland D, McDonald CR, Halgren E, Automated white-matter tractography using a probabilistic diffusion tensor atlas: Application to temporal lobe epilepsy. Hum Brain Mapp. 2009;30(5):1535–47. 18671230 10.1002/hbm.20619 PMC2754725

[65] Hagler DJ Jr, Ahmadi ME, Kuperman J, Holland D, McDonald CR, Halgren E, Automated white-matter tractography using a probabilistic diffusion tensor atlas: Application to temporal lobe epilepsy. Hum Brain Mapp. 2009;30(5):1535–47. 18671230 10.1002/hbm.20619 PMC2754725

[66] R Core Team. R: A language and environment for statistical computing. [Internet]. Vienna, Austria: R Foundation for Statistical Computing. 2019. https://www.R-project.org/

[67] Oksanen J, Simpson GL, Blanchet FG, Kindt R, Legendre P, Minchin PR vegan: Community Ecology Package [Internet]. 2022. https://CRAN.R-project.org/package=vegan

[68] Bates D, Maechler M, Bolker B, Walker S. Fitting Linear Mixed-Effects Models Using lme4. J Stat Softw. 2015;67(1):1–48.

[69] Ben-Shachar MS, Lüdecke D, Makowski D, effectsize. Estimation of effect size indices and standardized parameters. J Open Source Softw. 2020;5(56):2815.

[70] Markowski D, Ben-Shachar M, Lüdecke D, bayestestR. Describing Effects and their Uncertainty, Existence and Significance within the Bayesian Framework. J Open Source Softw. 2019;4(40):1541.

[71] Del Giudice M. Measuring sex differences and similarities. Gender and sexuality development: Contemporary theory and research. Springer; 2022. pp. 1–38.

[72] Fligner MA, Killeen TJ. Distribution-free two-sample tests for scale. J Am Stat Assoc. 1976;71(353):210–3.

[73] Cheng TW, Magis-Weinberg L, Guazzelli Williamson V, Ladouceur CD, Whittle SL, Herting MM, A Researcher’s Guide to the Measurement and Modeling of Puberty in the ABCD Study^®^ at Baseline. Front Endocrinol. 2021;12:608575. 10.3389/fendo.2021.608575 PMC8131843 34025573

[74] Herting MM, Uban KA, Gonzalez MR, Baker FC, Kan EC, Thompson WK, Correspondence Between Perceived Pubertal Development and Hormone Levels in 9–10 Year-Olds From the Adolescent Brain Cognitive Development Study. Front Endocrinol. 2020;11:549928. 10.3389/fendo.2020.549928 PMC7930488 33679599

[75] Petersen AC, Crockett L, Richards M, Boxer A. A self-report measure of pubertal status: Reliability, validity, and initial norms. J Youth Adolesc. 1988;17(2):117–33. 24277579 10.1007/BF01537962

[76] Thijssen S, Collins PF, Luciana M. Pubertal development mediates the association between family environment and brain structure and function in childhood. Dev Psychopathol 32(2):687–702. 31258099 10.1017/S0954579419000580 PMC7525116

[77] Nketia J, Amso D, Brito NH. Towards a more inclusive and equitable developmental cognitive neuroscience. Dev Cogn Neurosci. 2021;52:101014. 34571453 10.1016/j.dcn.2021.101014 PMC8476647

[78] Werchan DM, Amso D. A Novel Ecological Account of Prefrontal Cortex Functional Development. Psychol Rev. 2017;124(6):720–39. 29106267 10.1037/rev0000078 PMC5675045

[79] Liu S, Hou B, Zhang Y, Lin T, Fan X, You H, Inter-scanner reproducibility of brain volumetry: influence of automated brain segmentation software. BMC Neurosci. 2020;21(1):35. 32887546 10.1186/s12868-020-00585-1 PMC7472704

[80] Sanchis-Segura C, Ibañez-Gual MV, Aguirre N, Cruz-Gómez ÁJ, Forn C. Effects of different intracranial volume correction methods on univariate sex differences in grey matter volume and multivariate sex prediction. Sci Rep. 2020;10(1):12953. 32737332 10.1038/s41598-020-69361-9 PMC7395772

[81] Lebel C, Treit S, Beaulieu C. A review of diffusion MRI of typical white matter development from early childhood to young adulthood. NMR Biomed. 2019;32(4):e3778. 28886240 10.1002/nbm.3778

[82] Takao H, Hayashi N, Inano S, Ohtomo K. Effect of head size on diffusion tensor imaging. NeuroImage. 2011;57(3):958–67. 21605689 10.1016/j.neuroimage.2011.05.019

[83] Jamieson D, Shan Z, Sacks D, Boyes A, Lagopoulos J, Hermens DF. Investigating early adolescent sex differences in hippocampal and amygdala volumes, sleep quality and psychological distress. J Early Adolesc. 2023;43(3):360–78.

[84] Kurth F, Gaser C, Luders E. Development of sex differences in the human brain. Cogn Neurosci. 2020;1–8. 10.1080/17588928.2020.1800617 PMC8510853 32902364

[85] Peper JS, Brouwer RM, Schnack HG, van Baal GC, van Leeuwen M, van den Berg SM, Sex steroids and brain structure in pubertal boys and girls. Psychoneuroendocrinology. 2009;34(3):332–42. 18980810 10.1016/j.psyneuen.2008.09.012

[86] Raznahan A, Shaw P, Lalonde F, Stockman M, Wallace GL, Greenstein D, How Does Your Cortex Grow? J Neurosci. 2011;31(19):7174–7. 21562281 10.1523/JNEUROSCI.0054-11.2011 PMC3157294

[87] Forde NJ, Jeyachandra J, Joseph M, Jacobs GR, Dickie E, Satterthwaite TD, Sex Differences in Variability of Brain Structure Across the Lifespan. Cereb Cortex. 2020;30(10):5420–30. 32483605 10.1093/cercor/bhaa123 PMC7566684

[88] Ducharme S, Albaugh MD, Nguyen TV, Hudziak JJ, Mateos-Pérez JM, Labbe A, Trajectories of cortical thickness maturation in normal brain development — The importance of quality control procedures. NeuroImage. 2016;125:267–79. 26463175 10.1016/j.neuroimage.2015.10.010 PMC4691414

[89] Levenstein JM, Driver C, Boyes A, Parker M, Shan Z, Lagopoulos J, Sex differences in brain volumes and psychological distress: The first hundred brains cohort of the longitudinal adolescent brain study. Neuroimage Rep. 2023;3(2):100167. 10.1016/j.ynirp.2023.100167 PMC12172916 40568460

[90] Sussman D, Leung RC, Chakravarty MM, Lerch JP, Taylor MJ. The developing human brain: age-related changes in cortical, subcortical, and cerebellar anatomy. Brain Behav. 2016;6(4):e00457. 27066310 10.1002/brb3.457 PMC4802426

[91] Burger J Jr, Leadbetter M, Shaikh C. F. The Allometry of Brain Size in Mammals. 2018.

[92] Schoenemann P. Brain Size Scaling and Body Composition in Mammals. Brain Behav Evol. 2004;63:47–60. 14673198 10.1159/000073759

[93] Raznahan A, Parikshak NN, Chandran V, Blumenthal JD, Clasen LS, Alexander-Bloch AF, Sex-chromosome dosage effects on gene expression in humans. Proc Natl Acad Sci. 2018;115(28):7398–403. 29946024 10.1073/pnas.1802889115 PMC6048519

[94] Raznahan A, Disteche CM. X-chromosome regulation and sex differences in brain anatomy. Neurosci Biobehav Rev. 2021;120:28–47. 33171144 10.1016/j.neubiorev.2020.10.024 PMC7855816

[95] Herting MM, Gautam P, Spielberg JM, Dahl RE, Sowell ER. A Longitudinal Study: Changes in Cortical Thickness and Surface Area during Pubertal Maturation. Baud O, editor. PLOS ONE. 2015;10(3):e0119774. 25793383 10.1371/journal.pone.0119774 PMC4368209

[96] Savic I, Frisen L, Manzouri A, Nordenstrom A, Lindén Hirschberg A. Role of testosterone and Y chromosome genes for the masculinization of the human brain. Hum Brain Mapp. 2017;38(4):1801–14. 28070912 10.1002/hbm.23483 PMC6867124

[97] Laurent JS, Watts R, Adise S, Allgaier N, Chaarani B, Garavan H, Associations Among Body Mass Index, Cortical Thickness, and Executive Function in Children. JAMA Pediatr. 2020;174(2):170–7. 31816020 10.1001/jamapediatrics.2019.4708 PMC6902097

[98] Chaddock-Heyman L, Erickson KI, Kienzler C, King M, Pontifex MB, Raine LB, The Role of Aerobic Fitness in Cortical Thickness and Mathematics Achievement in Preadolescent Children. PLoS ONE. 2015;10(8):e0134115. 26267897 10.1371/journal.pone.0134115 PMC4534422

[99] Ruotsalainen I, Gorbach T, Perkola J, Renvall V, Syväoja HJ, Tammelin TH, Physical activity, aerobic fitness, and brain white matter: Their role for executive functions in adolescence. Dev Cogn Neurosci. 2020;42:100765. 32072938 10.1016/j.dcn.2020.100765 PMC7013351

[100] Breton E, Khundrakpam B, Jeon S, Evans A, Booij L. Cortical thickness and childhood eating behaviors: differences according to sex and age, and relevance for eating disorders. Eat Weight Disord. 2024;29(1):47. 39028377 10.1007/s40519-024-01675-3 PMC11271398

[101] Dick AS, Lopez DA, Watts AL, Heeringa S, Reuter C, Bartsch H, Meaningful associations in the adolescent brain cognitive development study. NeuroImage. 2021;239:118262. 34147629 10.1016/j.neuroimage.2021.118262 PMC8803401

[102] Warwick RM. Change in marine communities. 2001;Ch. 6.

[103] Arnold CE, Pilla R, Chaffin MK, Leatherwood JL, Wickersham TA, Callaway TR, The effects of signalment, diet, geographic location, season, and colitis associated with antimicrobial use or Salmonella infection on the fecal microbiome of horses. J Vet Intern Med. 2021;35(5):2437–48. 34268795 10.1111/jvim.16206 PMC8478058

[104] Clarke KR, Gorley RN. Primer v5: User manual/tutorial, primer e: Plymouth. Plymouth Mar Lab Plymouth UK; 2001.

[105] Davis Birch WA, Moura IB, Ewin DJ, Wilcox MH, Buckley AM, Culmer PR MiGut: A scalable in vitro platform for simulating the human gut microbiome—Development, validation and simulation of antibiotic-induced dysbiosis. Microb Biotechnol. 2023. 10.1111/1751-7915.14259 PMC10221534 37035991

[106] Cohen J. Statistical power analysis. Curr Dir Psychol Sci. 1992;1(3):98–101.

[107] Owens MM, Potter A, Hyatt CS, Albaugh M, Thompson WK, Jernigan T, Recalibrating expectations about effect size: A multi-method survey of effect sizes in the ABCD study. PLoS ONE. 2021;16(9):e0257535. 34555056 10.1371/journal.pone.0257535 PMC8460025

[108] Bigler RS, Signorella ML. Single-sex education: New perspectives and evidence on a continuing controversy. Sex Roles. 2011;65(9):659–69.

[109] Eliot L. Single-sex education and the brain. Sex Roles. 2013;69(7):363–81.

[110] Halpern DF, Eliot L, Bigler RS, Fabes RA, Hanish LD, Hyde J, The pseudoscience of single-sex schooling. Science. 2011;333(6050):1706–7. 21940879 10.1126/science.1205031

[111] Kennedy AD. The Brain-Sex Binary in Law: The influence of neurological theories of sex and gender on legal decision-making for trans and intersex minors. 2021.

[112] Re Alex: Hormonal Treatment for Gender Identity Dysphoria [Internet]. Cth. 2004 [cited 2023 Aug 18]. http://www6.austlii.edu.au/cgi-bin/viewdoc/au/cases/cth/FamCA/2004/297.html

[113] DiMarco M, Zhao H, Boulicault M, Richardson SS. Why sex as a biological variable conflicts with precision medicine initiatives. Cell Rep Med. 2022;3(4). 10.1016/j.xcrm.2022.100550 PMC9043982 35492240

[114] Miller VM, Rocca WA, Faubion SS. Sex Differences Research, Precision Medicine, and the Future of Women’s Health. J Womens Health. 2015;24(12):969–71. 10.1089/jwh.2015.5498 PMC4683561 26325362

[115] Richardson SS, Reiches M, Shattuck-Heidorn H, LaBonte ML, Consoli T. Focus on preclinical sex differences will not address women’s and men’s health disparities. Proc Natl Acad Sci. 2015;112(44):13419–20. 26534989 10.1073/pnas.1516958112 PMC4640753

[116] Eliot L, Ahmed A, Khan H, Patel J. Dump the dimorphism: Comprehensive synthesis of human brain studies reveals few male-female differences beyond size. Neurosci Biobehav Rev. 2021;125:667–97. 33621637 10.1016/j.neubiorev.2021.02.026

[117] van Anders SM. Gender/sex/ual diversity and biobehavioral research. Psychol Sex Orientat Gend Divers; 2022.

[118] Gagnier JJ, Morgenstern H. Misconceptions, Misuses, and Misinterpretations of P Values and Significance Testing. JBJS. 2017;99(18):1598. 10.2106/JBJS.16.01314 28926390

[119] Hoekstra R, Finch S, Kiers HA, Johnson A. Probability as certainty: Dichotomous thinking and the misuse of p values. Psychon Bull Rev. 2006;13:1033–7. 17484431 10.3758/bf03213921

[120] Bangdiwala SI. Understanding Significance and P-Values. Nepal J Epidemiol. 2016;6(1):522–4. 27152231 10.3126/nje.v6i1.14732 PMC4850233

[121] Mohajeri K, Mesgari M, Lee AS. When Statistical Significance Is Not Enough: Investigating Relevance, Practical Significance, and Statistical Significance. Manag Inf Syst Q. 2020;44(2):525–59.

